# PhyloJS: Bridging phylogenetics and web development with a JavaScript utility library

**DOI:** 10.1002/ece3.11603

**Published:** 2024-06-26

**Authors:** Leo A. Featherstone, Wytamma Wirth

**Affiliations:** ^1^ Peter Doherty Institute for Infection and Immunity University of Melbourne Melbourne Victoria Australia

**Keywords:** JavaScript, Phylogenetics, Web‐Development

## Abstract

There is an increasing number of libraries devoted to parsing, manipulating and visualising phylogenetic trees in JavaScript. Many of these libraries bundle tree manipulation with visualisation, but have limited ability to manipulate trees and lack detailed documentation. As the number of web‐based phylogenetic tools and the size of phylogenetics datasets increases, there is a need for a library that parses, writes and manipulates phylogenetic trees that is interoperable with other phylogenetic and data visualisation libraries. Here we introduce PhyloJS, a light zero‐dependency TypeScript and JavaScript library for reading, writing and manipulating phylogenetic trees. PhyloJS allows for modification of and data‐extraction from trees to integrate with other phylogenetics and data visualisation libraries. It can swiftly handle large trees, up to at least _10_^6^ tips in size, making it ideal for developing the next generation of more complex web‐based phylogenetics applications handling ever larger datasets. The PhyloJS source code is available on GitHub (https://github.com/clockor2/phylojs) and can be installed via npm with the command npm install phylojs. Extensive documentation is available at https://clockor2.github.io/phylojs/.

## Graphical abstract

A reliably JavaScript utility is lacking from the resources available to the computational biology community. PhyloJS fills this niche by providing a utility library distinct form visualisation, such that future phylogenetics web applications do not need to reinvent the wheel.

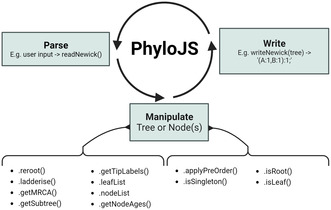

## 1 INTRODUCTION

Web‐based phylogenetics applications have become increasingly popular, concomitant with a general increase in web‐based bioinformatics applications. They offer notable benefit for the accessibility and scale of phylogenetic analysis. For example, Nextstrain makes the results of ongoing genomic epidemiology analyses accessible to non‐expert users through a web‐based interface (Hadfield et al., 2018). Similarly, Microreact provides a web‐based interface for the integrated visualisation of phylogenetic, spatial and epidemiological data (Argimón et al., 2016). The interactive exploration of trees with millions of tips has also been made possible with Taxonium (Sanderson, 2022). Other applications for visualising phylogenetic data include PhyD3 (Kreft et al., 2017), Phylo.io (Robinson et al., 2016) and IcyTree (Vaughan, 2017).

While many of the above applications excel at phylogenetic data visualisation and analysis, each implements its own code for parsing, writing and manipulating trees that is tightly coupled to the application itself, such that tree‐manipulating code is not separately available as a reusable package. In addition, other libraries that aim for broader utility integrate tree representation with the D3 visualisation library, inheriting dependencies and constraining their use in other applications (Bostock et al., 2011) (Table 1).

**TABLE 1:** A summary of phylogenetics libraries available on the npm registry.

	Version	Size (kb)	Dependencies	TypeScript	Networks	Newick	Nexus	phyloXML	NeXML	PhyJSON	Multiple trees	Reroot	Annotations	Extract clades
PhyloJS	1.5.10	15.1	0	Y	Y	Y	Y	Y	Y	Y	Y	Y	Y	Y
Phylotree (Shank et al., 2018)	1.4.0	238.8	11^a^	N	N	Y	Y	N	N	N	N	Y	Y	N
Phylocanvas (Abudahab et al., 2021)	2.8.1	53.6	0	N	N	Y	N	N	N	N	N	Y	N	N
Phylio	1.1.2	184	0	Y	N	Y	Y	Y	N	N	N	N	Y	N
PhyD3 (Kreft et al., 2017)	2.0.0	23.4	4^a^	Y	N	N	N	N	N	N	N	N	N	N
Newick‐js	1.2.1	5.1	0	Y	N	Y	N	N	N	N	N	N	N	N
newick‐reader	1.3.0	1.8	0	N	N	Y	N	N	N	N	N	N	N	N

*Note*: From the 57 libraries available as of early September 2023, we selected those which had reached version 1.0.0 and could parse a common tree format—Newick, Nexus, phyloXML or NeXML. The networks column refers to whether libraries are capable of parsing phylogenetic networks, which contain hybrid nodes. This table compares libraries in their ability to read, write and manipulate phylogenetic trees and networks. Many of the included libraries, such as phylocanvas, phylotree and phyD3, excel in visualisation beyond this. The size of each package is the minified bundle size.

^a^ Includes D3 as a dependency.

**TABLE 2:** A summary of each example and its potential or existing application in future or existing software.

Example	Application
Random Rerooting App	Any app requiring rerooting
Ladderising App	Improve visualisation for users
Newick Conversion App	Converting between tree formats
Tree statistics App	Calculate tree‐imbalance^a^
Fetching trees from URLs	Accessing online trees (e.g. UShER output (Turakhia et al., 2021))
Root‐to‐tip Regression	Clockor2^b^
Pruning and Grafting	Removing outlier clades, topological operators in MCMC
Extracting Clades	Isolating clades of epidemiological interest
Modifying Annotations	Linking metadata
Annotations and Tree Traversal	Tree traversal (e.g. best fitting root in TreeTime (Sagulenko et al., 2018))
Arrays of Trees	Handle Posterior distributions of trees

^a^ Tree statistics including tree length, the Gamma statistic (Pybus & Harvey, 2000), Sackin Index and Colless Imbalance Index (Fischer et al., 2023).

^b^ The root‐to‐tip regression example is a significant simplification of Clockor2, which makes extensive use of PhyloJS.

Overall, as of early September 2023 there were 57 packages matching the search term ‘phylogenetic’ in the npm registry (the main repository for JavaScript packages, akin to CRAN and PyPI for R and Python packages) from the last 10 years (Table 1). Of these, the majority became available in the last 5 years. All of these are either primarily devoted to visualisation, or offer limited ability to manipulate trees to the extent available in the state‐of‐the‐art libraries of other ecosystems such as ape or treeio in R; dendroPy or ETE3 in python; and PhyloNetworks in julia (Huerta‐Cepas et al., 2016; Paradis & Schliep, 2019; Solís‐Lemus et al., 2017; Yu et al., 2017).

Although visualisation is essential because phylogenetic trees are inherently visual models, the large number of visualisation libraries has created a niche for a library solely devoted to manipulating phylogenetic trees. Moreover, such a library further enables phylogenetic computation in client‐side applications, where all computation is done in the browser without need for a server. This has notable benefits for data security and accessibility, especially for sensitive data in genomic epidemiology since the data never leaves the user's computer. In conjunction, visualisation and manipulation‐oriented libraries will allow developers to produce applications with both high quality visualisation and greater functionality.

Here we present, PhyloJS, a well‐documented, standardised and standalone JavaScript library for parsing, writing and manipulating phylogenetic trees to develop novel phylogenetics web‐based applications with an emphasis on client‐side computation. PhyloJS focuses on tree manipulation rather than visualisation and can efficiently interface other phylogenetic and data visualisation libraries, such as phylocanvas, phylotree or plotly (Abudahab et al., 2021; Plotly‐Technologies‐Inc, 2015; Shank et al., 2018).

## 2 IMPLEMENTATION AND USAGE

A detailed application programming interface (API) for PhyloJS documenting all functions, methods and classes can be found at https://clockor2.github.io/phylojs/.

**FIGURE 1:**
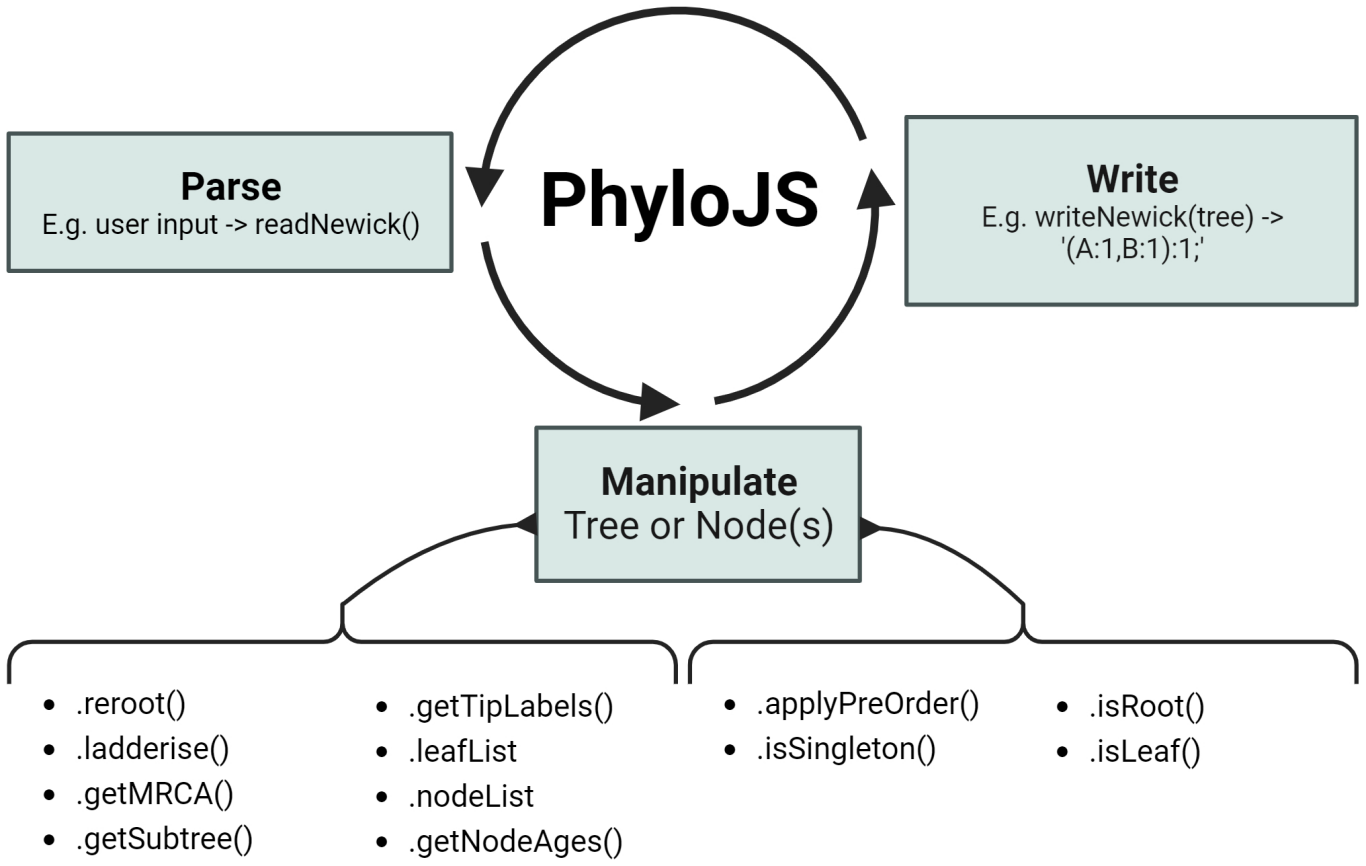
Conceptual figure of PhyloJS' utility, with a non‐exhaustive list of functions and methods for parsing and manipulating trees and nodes. PhyloJS parses trees, such as from user input and other libraries, and provides a utility library to operate on the tree and return it for visualisation, user interaction and processing via other libraries.

The representation, parsing and writing of trees in PhyloJS is based on the algorithms used in IcyTree, a well‐established client‐side visualisation tool for phylogenetic trees (Vaughan, 2017). PhyloJS is written in TypeScript, supporting type‐safe development and, therefore, is ideal for debugging larger applications. Overall, it offers a package that will be familiar to those who have used popular phylogenetic utility libraries in other languages such as ape (Paradis & Schliep, 2019) in R and DendroPy (Sukumaran & Holder, 2010) in Python (Figure 1).

PhyloJS includes two classes: Node and Tree (Figure 1). The Node class includes properties for branch length (branchLength) leading into the node, as well as descending and ancestral nodes (children and parent, respectively). Child and parent properties facilitate the nesting of nodes, wherein a node can have any number of descending child nodes and up to one parent (zero for the root). Node objects also store annotations via the annotation property, which is itself an object storing key‐value pairs of annotations. Hybrid nodes in phylogenetic networks are also denoted with a Boolean property (isHybrid = TRUE).

The Tree class is a wrapper of the Node class. It contains the root node at the highest level, and each subsequent node nested within it. It includes getters for internal nodes and tips (.nodeList and .leafList, respectively), which return arrays of Node objects (a leaf is a node object without any child nodes). The Tree class includes many other methods for manipulating trees, referenced in the following sections and examples.

### 2.1 Parsing

PhyloJS includes functions to read annotated and un‐annotated trees from all common formats (Newick, Nexus, PhyloXML, NeXML and PhyJSON), (Boettiger et al., 2016; Cardona et al., 2008; Han & Zmasek, 2009; Maddison et al., 1997; Ronquist et al., 2021). PhyloJS can also parse lists of trees separated by the newline character in formats additional to Nexus (e.g. readTreesFromNewick(), readTreesFromPhyloXML()) to arrays of Tree objects. This is useful for efficiently manipulating multiple trees at once using array methods in JavaScript (e.g. TreesArray.map(tree = > tree.ladderise())). Finally, PhyloJS includes a general function read(), that accepts strings and a schema argument to select a particular file format. It returns a Tree array (Tree[]) in all cases and is useful for applications with multiple input file formats.

### 2.2 Writing

PhyloJS writes individual trees to Newick and Nexus formats (writeNewick(), writeNexus()). Both functions also accept a callback for including annotations. The default behaviour is not to annotate and users can supply their own annotator or use the inbuilt beastAnnotation() or nhxAnnotation() functions to write annotations in the common formats used in BEAST or NHX (Bouckaert et al., 2019; Suchard et al., 2018). If users wish to write from an array of trees to one string, then writing functions can be applied via a JavaScript array method (e.g. TreesArray.map(tree = > writeNewick(tree)).join(’\n’)). The branch length property (.branchLength) for each node can be undefined, and all tree‐writing functions omit undefined branch lengths when writing. Last, PhyloJS does not automatically resolve polytomies and thus can write and parse multifurcating trees.

### 2.3 Manipulating

The Tree and Node classes include a number of methods that are unique among JavaScript libraries and or useful for manipulating trees. For topological manipulation, the Tree class includes methods for rerooting (.reroot()), ladderisation (.ladderise()) and extraction of subtrees (.getClade()). It also includes methods for accessing common ancestors (.getMRCA()).

The Node class also includes methods to add and remove children (.addChild() and .removeChild()), get ancestral nodes (.getAncestors()) and apply a function via a pre‐order and post‐order traversal of nested nodes (.applyPreOrder() and .applyPostOrder()). Branch length modifications can be applied directly to the .branchLength property of each node.

The Tree class also includes several convenience methods that help to extract data from the trees. For example, (.getRTTDist()) returns root‐to‐tip distances for each tip and (.getBranchLengths()) returns all branch lengths. For example, the root‐to‐tip regression tool Clockor2 (clockor2.github.io) uses these methods internally (Featherstone et al., 2024).

### 2.4 Benchmarking

**FIGURE 2:**
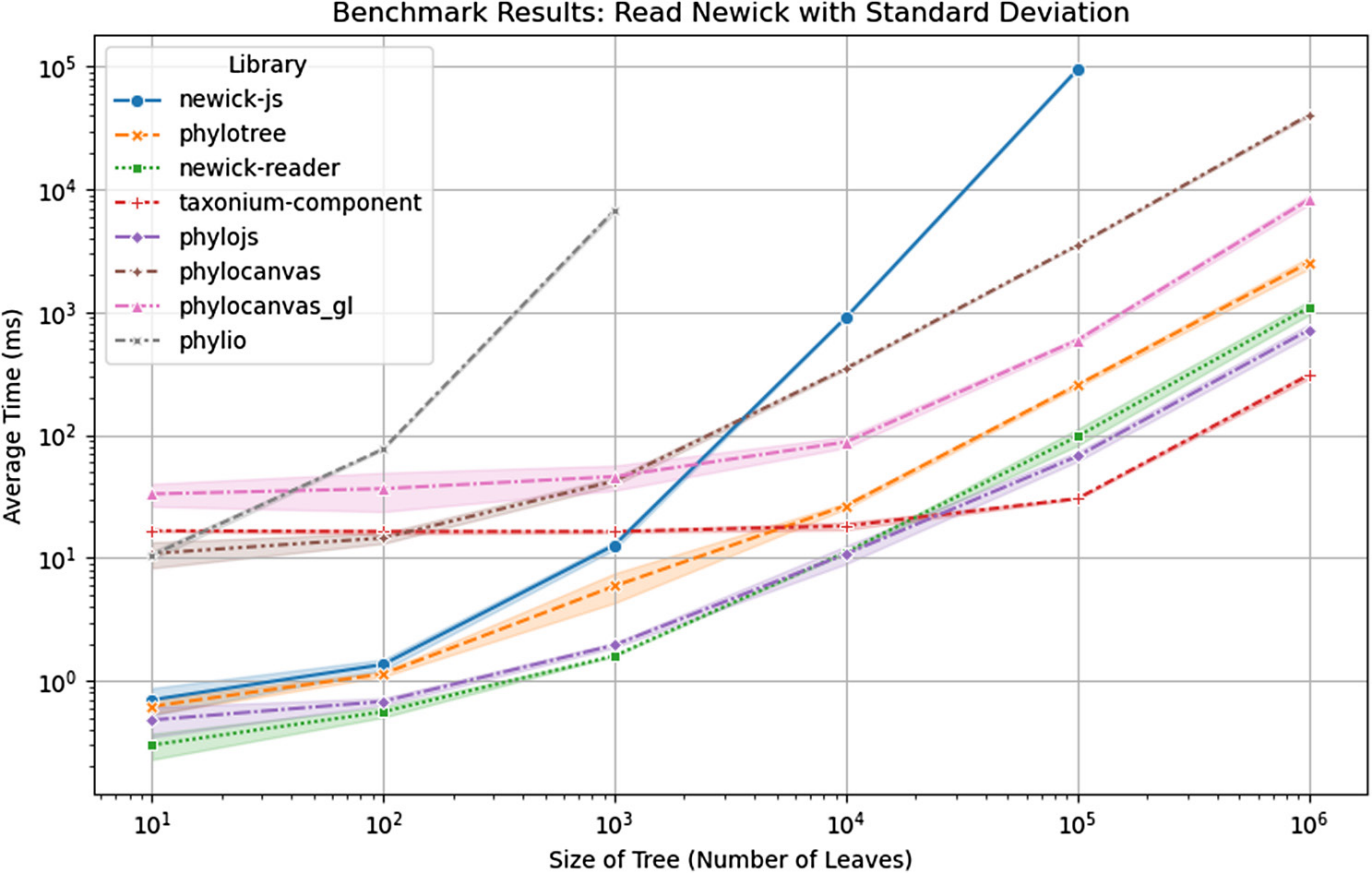
A comparison of time taken in milliseconds to parse Newick trees of increasing size. Colour corresponds to each phylogenetics library and shading represents the standard deviation of time taken to parse a tree of size n across 5 duplicates. PhyloJS is among the fastest, with a minor performance loss compared to newick‐reader due to additional logic for parsing annotations and hybrid nodes. Taxonium, though not formally a library for manipulating phylogenetic trees, is included as a comparison because it is optimised for parsing large trees. Reads that took longer than 100 s to complete were cancelled.

We benchmarked PhyloJS against other libraries in Table 1 by parsing simulated Newick trees with 10^1^ to 10^6^ tips. Parsing Newick trees presents a task common to all phylogenetics libraries, and we note that methods on the Tree class remained fast for the largest trees. PhyloJS was among the fastest, with only a fractional delay compared to newick‐reader (<1(ms)) for trees with fewer than 10^4^ tips. This was probably due to additional logic in PhyloJS' Newick parser, which accounts for annotations and hybrid nodes and is lacking in newick‐reader (Figure 2). Taxonum‐component, though not a library for manipulating phylogenetic trees, is included as a comparison because it presents the state‐of‐the‐art parser optimised for the largest phylogenetic trees Sanderson (2022). For trees with more than 10^4^ tips, PhyloJS is second to Taxonium in speed.

Both PhyloJS and Taxonium incorporate a parser that utilises a stack to read Newick strings, based on the parser in jstreeview (https://github.com/lh3/jstreeview/tree/main). We found this approach conferred a notable speedup in comparison to recursive approaches and the capacity to parse much larger trees.

## 3 EXAMPLES AND DOCUMENTATION

Documentation for PhyloJS includes an API reference and several interactive examples at https://clockor2.github.io/phylojs/. Most examples demonstrate how to build small applications performing functions such as finding the most recent common ancestor for sets of tips, root‐to‐tip regression, rerooting, interfacing with visualisation libraries, handling multiple trees concurrently, modifying annotations, pruning and grafting clades and calculating summary statistics. Some examples also demonstrate how developers may parse trees from user input, including from URLs. Example applications also demonstrate how PhyloJS can be used to interface with visualisation libraries, such as phylocanvas (Abudahab et al., 2021) (Table 2).

Below, we include examples highlighting applications of PhyloJS, and how the code looks in practice. The final two examples demonstrate working with arrays of trees and annotating nodes with summary statistics. Together, they demonstrate PhyloJS’ ability to concurrently process trees, modify annotations, as well as modify topology and branch lengths. We urge readers wishing to run the code to view these as interactive examples at (https://clockor2.github.io/phylojs/).

### 3.1 Clockor2: A larger scale example

PhyloJS was initially developed to support phylogenetics web applications that incorporate analysis and visualisation without the need for server side computation. Clockor2 (https://clockor2.github.io/) is a key example, performing root‐to‐tip regression with strict local molecular clocks in the browser using PhyloJS. Here, PhyloJS provides all of the functionality to parse, manipulate and analyse trees including root‐to tip regression. All operations make use of PhyloJS, which parses and returns trees in Newick format for visualisation using phylocanvas and Plotly, or for download (Abudahab et al., 2021; Plotly‐Technologies‐Inc, 2015).

### 3.2 Interfacing with visualisation libraries

For a minimal example of interfacing PhyloJS with a visualisation library, we direct readers to the visualisation example https://clockor2.github.io/phylojs/examples/visualise/, which uses PhyloJS to randomly reroot a tree and phylocanvas for visualisation (Abudahab et al., 2021).

### 3.3 Working with arrays of trees

In the following example, we parse two trees written in Newick format to an array of Tree objects using readTreesFromNewick(). For both trees, all branch lengths are equal to 1. We arbitrarily reroot each tree at the 4th node, ladderise them and randomly rescale each branch length. We finally write the resulting trees to Newick format. This example demonstrates how users can manipulate multiple trees concurrently. // Using two small trees with 3 tips and all branch lengths set to 1. const inNewick = ’ (( A :1 , B : 1 ) : 1 , C : 1 ) ; \n (( A :1 , B : 1 ) : 1 , C : 1 ) ; ’ l e t trees = readTreesFromNewick ( inNewick  ) ; // Operate on trees using array methods. Reroot , ladderise , and scale branch lengths randomly trees. forEach ( t => t. reroot ( t. nodeList [4] ) ) // ar b i t r a r i l y to 4 th node trees. forEach ( t => t. ladderise ()) trees. forEach ( t => t. nodeList. forEach ( n => n. branchLength !== undefined ? n. branchLength *= Math. floor (10* Math. random () + 1) : 0 )) // write output l e t outNewick = trees. map ( t => writeNewick ( t ) ). join ( ’ \n ’ ) console. log ( outNewick ) // (" C ":1.5 ,(" B ":5 ," A ":6):4.5):0.0; // (" C ":1 ,(" B ":8 ," A ":16):4):0.0;


### 3.4 Internal to external branch length ratio annotation

Here, we demonstrate how to calculate the internal to external branch length ratio (IE ratio hereon) for clades descending from each internal node of a tree. We then add the statistic as a node annotation. This sort of program could, for example, be implemented as part of an application to visualise parts of a tree that drive values of summary statistics the most. // IE ratio function function getIERatio ( tree : Tree ) : number { l e t sumInternal : number = 0. 0 ; l e t sumExternal : number = 0. 0 ; for ( l e t i =0; i < tree. nodeList. length ; i ++) { if ( tree. nodeList [ i ]. branchLength !== undefined ){ if ( tree. nodeList [ i ]. isLeaf ()) { sumExternal += tree. nodeList [ i ]. branchLength } else { sumInternal += tree. nodeList [ i ]. branchLength } } } return sumInternal / sumExternal ; } l e t nwk = ‘(( a :2 , b : 2 ) : 1 , ( c :1 , d : 1 ) : 4 ) ; ‘ l e t tree = readNewick ( nwk ) // get IE ratio for subtrees descending from each node , except tips tree. root. applyPreOrder (( node : Node ) => { if ( ! node. isLeaf ()) { l e t ieRatio = getIERatio ( tree. getSubtree ( node )) node. annotation = { ieRatio : ieRatio. toFixed (2)} } }); // Expect annotations in newick with ‘ true ’ flag console. log ( writeNewick ( tree , beastAnnotation )) // Returns : // ((" a ":2 ," b ":2[&" ieRatio "="0.25"]:1(" c ":1 ," d ":1[" ieRatio "="2.00"]:4[&" ieRatio "="0.83"]:0.0;


## 4 CONCLUSIONS

PhyloJS helps to fill the niche for a general phylogenetics utility library in the JavaScript ecosystem. Among the diversity of libraries providing specialised functionality, particularly for visualisation, PhyloJS serves as foundation for manipulating trees and inter‐operating with other libraries to build large phylogenetics applications. It offers a mix of scale and flexibility, handling phylogenetic trees and networks from many formats up to at least 10^6^ tips in size, and including diversity of methods with which to modify and analyse trees. Together with its extensive documentation, it will help developers to build the next generation of web‐based phylogenetics applications without reinventing the wheel.

## AUTHOR CONTRIBUTIONS

**Leo A. Featherstone:** Conceptualization (equal); software (equal); writing – original draft (lead); writing – review and editing (lead). **Wytamma Wirth:** Conceptualization (equal); software (lead); supervision (lead); writing – original draft (supporting); writing – review and editing (supporting).

## CONFLICT OF INTEREST STATEMENT

LAF and WW declare no conflicts of interest.

## Data Availability

PhyloJS is open‐source and freely available at https://github.com/clockor2/phylojs. PhyloJS has a liberal licence (GNU General Public Licence v3.0) allowing for commercial use and derivative works. PhyloJS can be installed via npm with the command npm instal phylojs or included via a script tag using CDN URL https://unpkg.com/phylojs@latest/lib/dist/phylojs.min.js. All documentation is available at https://clockor2.github.io/phylojs/.
